# Supplementary material for: Helminth infection modulates systemic pro-inflammatory cytokines and chemokines implicated in type 2 diabetes mellitus pathogenesis
Source: PLoS Negl Trop Dis. 2020 Mar 3;14(3):e0008101. doi: 10.1371/journal.pntd.0008101 (PMC7069638; doi:10.1371/journal.pntd.0008101)
Supplement: Attachment — Submitted filename: Reply to Reviewer comments.docx [file pntd.0008101.s001.docx]

Dear Dr. Babu:

Thank you very much for submitting your manuscript "Helminth infection modulates systemic pro-inflammatory cytokines and chemokines implicated in Type 2 diabetes mellitus pathogenesis" (#PNTD-D-19-01779) for review by PLOS Neglected Tropical Diseases. Your manuscript was fully evaluated at the editorial level and by independent peer reviewers. The reviewers appreciated the attention to an important problem, but raised some substantial concerns about the manuscript as it currently stands. These issues must be addressed before we would be willing to consider a revised version of your study. We cannot, of course, promise publication at that time.

We therefore ask you to modify the manuscript according to the review recommendations before we can consider your manuscript for acceptance. Your revisions should address the specific points made by each reviewer. 

When you are ready to resubmit, please be prepared to upload the following:

(1) A letter containing a detailed list of your responses to the review comments and a description of the changes you have made in the manuscript.

(2) Two versions of the manuscript: one with either highlights or tracked changes denoting where the text has been changed (uploaded as a "Revised Article with Changes Highlighted" file); the other a clean version (uploaded as the article file).

(3) If available, a striking still image (a new image if one is available or an existing one from within your manuscript). If your manuscript is accepted for publication, this image may be featured on our website. Images should ideally be high resolution, eye-catching, single panel images; where one is available, please use 'add file' at the time of resubmission and select 'striking image' as the file type. 

Please provide a short caption, including credits, uploaded as a separate "Other" file. If your image is from someone other than yourself, please ensure that the artist has read and agreed to the terms and conditions of the Creative Commons Attribution License at http://journals.plos.org/plosntds/s/content-license (NOTE: we cannot publish copyrighted images). 

(4) If applicable, we encourage you to add a list of accession numbers/ID numbers for genes and proteins mentioned in the text (these should be listed as a paragraph at the end of the manuscript). You can supply accession numbers for any database, so long as the database is publicly accessible and stable. Examples include LocusLink and SwissProt.

(5) To enhance the reproducibility of your results, we recommend that you deposit your laboratory protocols in protocols.io, where a protocol can be assigned its own identifier (DOI) such that it can be cited independently in the future. For instructions see http://journals.plos.org/plosntds/s/submission-guidelines#loc-methods

Please note while forming your response, if your article is accepted, you may have the opportunity to make the peer review history publicly available. The record will include editor decision letters (with reviews) and your responses to reviewer comments. If eligible, we will contact you to opt in or out.

While revising your submission, please upload your figure files to the Preflight Analysis and Conversion Engine (PACE) digital diagnostic tool, https://pacev2.apexcovantage.com/ PACE helps ensure that figures meet PLOS requirements. To use PACE, you must first register as a user. Then, login and navigate to the UPLOAD tab, where you will find detailed instructions on how to use the tool. If you encounter any issues or have any questions when using PACE, please email us at figures@plos.org.

We hope to receive your revised manuscript by Feb 17 2020 11:59PM. If you anticipate any delay in its return, we ask that you let us know the expected resubmission date by replying to this email.

To submit a revision, go to https://www.editorialmanager.com/pntd/ and log in as an Author. You will see a menu item call Submission Needing Revision. You will find your submission record there. 

Sincerely,

Mathieu Nacher
Guest Editor
PLOS Neglected Tropical Diseases

Maria Periago
Deputy Editor
PLOS Neglected Tropical Diseases

***********************


Reviewer's Responses to Questions

Key Review Criteria Required for Acceptance?


As you describe the new analyses required for acceptance, please consider the following:


Methods


-Are the objectives of the study clearly articulated with a clear testable hypothesis stated?

-Is the study design appropriate to address the stated objectives?

-Is the population clearly described and appropriate for the hypothesis being tested?

-Is the sample size sufficient to ensure adequate power to address the hypothesis being tested?

-Were correct statistical analysis used to support conclusions?

-Are there concerns about ethical or regulatory requirements being met?


Reviewer #1: Do the author have data on CRP IL-1Ra? This inflammation markers are very robust and changes would support authors hypothesis

**Response:** Thanks for your suggestion. In the Current study, we wanted to examine the systemic responses of cytokines and chemokines in T2DM with or without helminth infection. Now we have shown the data on IL-1Ra in figures 1 and 3. We do not have data on CRP.

Reviewer #2: The work developed demonstrates that Ss+ individuals exhibited significantly diminished levels of the proinflammatory cytokines and chemokines. Anthelmintic treatment resulted in increased levels of all of the cytokines and chemokines, being a work of great relevance. The same research group published previous work with the same samples studied in: Clin Infect Dis. 2019 Aug 15; 69(4): 697–704. Metabolic Consequences of Concomitant Strongyloides stercoralis Infection in Patients with Type 2 Diabetes Mellitus with equal design and only differentiating in the studied cytokines. I suggest the author submit the article as short communication to show the other data found.

-Are the objectives of the study clearly articulated with a clear testable hypothesis stated? YES

-Is the study design appropriate to address the stated objectives? YES

- Is the population clearly described and appropriate for the hypothesis being tested? NO

- Is the sample size sufficient to ensure adequate power to address the hypothesis being tested? NO

- Were correct statistical analysis used to support conclusions? NO

- -Are there concerns about ethical or regulatory requirements being met? YES

- How was sample calculated? How were participants selected?

**Response:** Sample size calculation was done to detect a significant difference (p<0.05) among the cytokines and chemokines based on preliminary analysis between the two groups. We determined that we needed 58 individuals in each group to detect this difference with a power of 90% and a Type I error of 5%. This is now mentioned in the Methods.

- What parasitological method is used? How many samples were analyzed? What other methods were used to exclude other helminths? describe in more detail the methods used.

**Response:** *Strongyloides stercoralis* (*Ss*) infection was diagnosed by the presence of IgG antibodies to the recombinant NIE antigen as described previously (Bisoffi Z et al PLoS Negl Trop Dis. 2014, Buonfrate D et al, PLoS Negl Trop Dis. 2015). A single stool sample was obtained and examined for intestinal helminth infection by Kato-Katz technique. Stool samples found to be negative for other intestinal helminths by stool microscopy and positive for *Ss* infection by serology were then subjected to specialized stool examination with nutrient agar plate cultures. Filarial infection was excluded in all study participants by virtue of being negative in tests for circulating filarial antigen. All subjects showed no evidence of other soil-transmitted helminths. This is now mentioned in the Material and Methods.

- The participants with strongyloides had no other helminths? Other studies verify the influence of other helminths and protozoa in dm2, being important to exclude other parasites or to include in the analyzes.

**Response:** The study participants had no other helminth infection other than *Strongyloides stercoralis.* We did not test for enteric protozoa. This is now mentioned in the Material and Methods.

- Was only fasting glucose and HbA1c used to confirm diabetes? Why was the glucose tolerance test not performed? may have samples that are intolerant that they could have failed to include using fasting glucose alone.

**Response:** All of them were newly diagnosed as type 2 diabetes. Since it is field based study, we had limited resources and we were not able to perform glucose tolerance test at the time of screening. Hence, we performed fasting blood glucose, HbA1c and Random blood glucose to know their DM status. Fasting blood glucose and HbA1c are sufficient for diagnosing DM, according to the ADA criteria.

- How dm1 and type 2 was differentiated? how can you be sure to be type 2? Clinical signs were considered to differentiate between dm1 and dm2.

**Response:** T1DM was unlikely on the basis of age. The diabetic individuals were 22 or older.

- What is the rationale for hematological and other biochemical analyzes of the project? what purpose? This data was not used for anything.

**Response:** We have removed the reference to hematological parameters. Other biochemical parameters were measured to examine renal and liver function in the diabetic individuals.

- Justify the statistical analyzes studied at work. why was it used? why no logistic regression was performed to evaluate the effect of Strongyloides on dm2

**Response:** We have performed standard statistical analysis for non-parametric values. The data was tested using the Shapiro-Wilk normality test and found to be non-normally distributed. Hence, for the comparison of two group (i.e. Ss+ and Ss-) a non-parametric test Mann-Whitney was used. PCA was used for the comparison and association between the clustered cytokines.

The logistic regression is not suitable for this data to predict the group differences (i.e. *Ss+* and *Ss-*) because of the multicollinearity between most of the key variables. Those variables significantly different by Mann-Whitney test were also significantly different by the univariate logistics regression model. This is now mentioned in the Methods.

- For the analysis of the comparison of the groups in relation to the dosages, how was the comparison made? using average or median? looking at the figures some analysis seems that the confidence interval overlaps between the groups and would not have significant difference.

**Response:** Geometric means were used as the measure of central tendency.

- Why was no analysis of clustered cytokines performed? What relationship between them?

**Response:** We have performed PCA analysis in this study. This is shown in Figure 5.

Reviewer #3: -Are the objectives of the study clearly articulated with a clear testable hypothesis stated?
YES
-Is the study design appropriate to address the stated objectives?
YES
-Is the population clearly described and appropriate for the hypothesis being tested?
YES
-Is the sample size sufficient to ensure adequate power to address the hypothesis being tested?
YES
-Were correct statistical analysis used to support conclusions?
YES
-Are there concerns about ethical or regulatory requirements being met?
YES

--------------------

Results


-Does the analysis presented match the analysis plan?

-Are the results clearly and completely presented?

-Are the figures (Tables, Images) of sufficient quality for clarity?


Reviewer #1: Figure 3 and 4 are visually not well presented. It would be helpful to see the mean values of pre and post, and may be have different colors for changes going up or down

**Response:** Thanks for your comments. Now we included the arrow mark to show the changes.

Reviewer #2: -Does the analysis presented match the analysis plan? NO

- -Are the results clearly and completely presented? NO

- -Are the figures (Tables, Images) of sufficient quality for clarity? YES

Reviewer #3: -Does the analysis presented match the analysis plan?
YES
-Are the results clearly and completely presented?
YES
-Are the figures (Tables, Images) of sufficient quality for clarity?
YES

--------------------

Conclusions


-Are the conclusions supported by the data presented?

-Are the limitations of analysis clearly described?

-Do the authors discuss how these data can be helpful to advance our understanding of the topic under study?

-Is public health relevance addressed?


Reviewer #1: Yes

Reviewer #2: -Are the conclusions supported by the data presented? NO

-Are the limitations of analysis clearly described? NO

**Response:** We have now added the following: The limitations of the study include the absence of oral glucose tolerance testing for diabetes, the moderate sample size and the absence of testing for protozoa.


-Do the authors discuss how these data can be helpful to advance our understanding of the topic under study? YES

-Is public health relevance addressed? NO

**Response:** We have mentioned that: Nevertheless, our study offers novel insights into the immunological interactions between helminth infections and metabolic disorders. In summary, our study demonstrates that *Ss* infection may provide a degree of protection from the pathology associated with T2DM by modulating the surrounding cytokine and chemokine milieu. Our data also suggest that helminth derived molecules or even helminth infection (per se) could offer novel therapeutic approaches to treating inflammatory metabolic diseases.

Reviewer #3: -Are the conclusions supported by the data presented?
YES, but the author did not measure any anti-inflammatory markers
-Are the limitations of analysis clearly described?
NO, the author should add the limitations of thi study

**Response:** In our previous study, “Clin Infect Dis. 2019 Aug 15; 69(4): 697–704. Metabolic Consequences of Concomitant Strongyloides stercoralis Infection in Patients with Type 2 Diabetes Mellitus”, we have shown Th1, Th2, Th17 and regulatory cytokine responses. Hence, we have not measured them in this current study.

We have now added the following: The limitations of the study include the absence of oral glucose tolerance testing for diabetes, the moderate sample size and the absence of testing for protozoa.

-Do the authors discuss how these data can be helpful to advance our understanding of the topic under study?
YES
-Is public health relevance addressed?
YES, partially.

--------------------

Editorial and Data Presentation Modifications?


Use this section for editorial suggestions as well as relatively minor modifications of existing data that would enhance clarity. If the only modifications needed are minor and/or editorial, you may wish to recommend “Minor Revision” or “Accept”. 

Reviewer #1: See above my suggestions which I consider as minor

Reviewer #2: (No Response)

Reviewer #3: Method Section:
For the determination of T2DM status, the author used HbA1c and Random Blood Glucose. If possible, the author should also describe other factors that might affect HbA1c level in the study population eg anemia, etc.

**Response:** We have excluded “those who have iron deficiency, anemia, alcoholism, chronic renal failure, hyperbilirubinemia and those who taking large doses of aspirin”.

For the measurement of cytokines level, the author should describe whether the measurements were done in paired samples (SS pos and SS neg side by side for each sample)?

**Response:** We measured the samples Ss positive and Ss negative side by side for each sample.

The author should also describe about other factors that might affect inflammatory markers, eg infections, medications, etc -- and whether this has been addressed by excluding those subjects or adjust for these factors in the analysis

**Response:** Individuals with any previous history of helminth infection or previous anthelmintic treatment or HIV infections, individuals those who have iron deficiency, anemia, alcoholism, chronic renal failure, hyperbilirubinemia and those taking large doses of aspirin were excluded from the study.
--------------------

Summary and General Comments


Use this section to provide overall comments, discuss strengths/weaknesses of the study, novelty, significance, general execution and scholarship. You may also include additional comments for the author, including concerns about dual publication, research ethics, or publication ethics. If requesting major revision, please articulate the new experiments that are needed.

Reviewer #1: This is a very interesting observational study, which may be a starting point for further studies and drug development. The hypothesis is clear, the data are solid and the manuscript is well written. The major limitation is that the study is purely correlative, but it does not take away from its originality.

Reviewer #2: (No Response)

Reviewer #3: This is a well written article. The author describes the fact that helminth infections could modulate systemic pro-inflammatory cytokines and chemokines in newly diagnosed diabetes.

However, several issues need to be addressed.

--------------------

PLOS authors have the option to publish the peer review history of their article ([what does this mean?](https://journals.plos.org/plosntds/s/editorial-and-peer-review-process#loc-peer-review-history)). If published, this will include your full peer review and any attached files.


If you choose “no”, your identity will remain anonymous but your review may still be made public.


Do you want your identity to be public for this peer review? For information about this choice, including consent withdrawal, please see our [Privacy Policy](https://www.plos.org/privacy-policy).

Reviewer #1: Yes: Marc Donath

Reviewer #2: No

Reviewer #3: No

__________________________________________________
In compliance with data protection regulations, you may request that we remove your personal registration details at any time. (Use the following URL: https://www.editorialmanager.com/pntd/login.asp?a=r). Please contact the publication office if you have any questions.
